# Bridging crisis recovery and long-term transformation of the health system in Lebanon: evidence from key informant interviews and global lessons

**DOI:** 10.1186/s12913-026-14630-y

**Published:** 2026-04-27

**Authors:** Jade Khalife, Alissar Rady, Hikma Shoaib, Aya Harb, Hilda Harb, Rasha Hamra, Walid Ammar

**Affiliations:** 1 Higher Institute of Public Health, Faculty of Medicine, Saint Joseph University of Beirut, Beirut, Lebanon; 2 Country Office for Lebanon, World Health Organization, Beirut, Lebanon; 3 Ministry of Public Health, Beirut, Lebanon

**Keywords:** Strategy, Health system, Crisis, Reform, Emergency, Governance, Financing, Accountability, Corruption, Workforce, People-centered, Infrastructure, Service delivery, Lebanon

## Abstract

**Background:**

Since late 2019, Lebanon’s health system has faced immense strain an unprecedented financial and economic crisis, aggravated by the COVID-19 pandemic, and the Beirut Port explosion which damaged or destroyed a third of the capital, Beirut. This has created an urgent need for reform, heightened by the subsequent conflict (2023–2026) and its additional toll on health infrastructure and personnel.

**Methods:**

A purposive sample of 27 key informants was chosen, based on their expertise in public health, hospital management, healthcare financing, and policy-making. Informants included policymakers, academics, directors of hospitals and primary care centers, and health leaders. This study was conducted in 2022. Semi-structured interviews were held with informants, and thematic analysis was used to analyze the data collected.

**Results:**

This study identified six themes and 26 subthemes. The analysis reveals the challenges in health reform, financing, healthcare access and delivery, health personnel, information systems, and transitioning to a people-centered health system. These challenges are attributable to key challenges including sectarian clientelism driving political interference, accountability deficits, financial system failure, economic collapse, fragmented financing and services, personnel crisis and infrastructure gaps.

**Conclusion:**

There is an urgent need for transformative reforms for Lebanon’s health system. A list of 22 impactful and scalable recommendations is proposed, including foundational measures and immediate stabilization measures. Foundational measures include unifying public payers (harmonization initially), creating a National Health Council, defining clear roles for public and private hospitals, and improving personnel working conditions. Immediate measures include adopting electronic prescriptions and telemedicine licenses; centralizing emergency dispatch, creating a National Patient Association, and implementing national electronic health records. Achieving such reforms hinges on separating health governance from political interference and empowering technical leadership.

**Supplementary Information:**

The online version contains supplementary material available at 10.1186/s12913-026-14630-y.

## Introduction

Lebanon has witnessed high political instability over the past five decades. It emerged from the 1975–1990 civil war facing multi-faceted challenges, including a crippled infrastructure, soaring poverty, and a debilitated health system. Despite these challenges - compounded by concurrent political interference and clientelism - the next three decades saw considerable advancements in achieving health goals and rebuilding the Lebanese health system [1]. These are largely attributable to improved socioeconomic conditions and the collaborative governance approach led by the technical leadership of the Ministry of Public Health (MoPH), together with partner organizations [1]. Notable achievements included meeting the fourth and fifth Millennium Development Goals (MDGs) for reducing maternal and child mortality, respectively, alongside increasing life expectancy - placing Lebanon several years above the regional average [2–4]. Additionally, Lebanon also ranked in the top quintile globally for healthcare access and outcomes [5, 6]. Remarkably, the health system withstood the turmoil that began in 2005, enduring a series of political assassinations, a devastating military attack in 2006, frequent political deadlocks, and the subsequent massive influx of refugees starting in 2013 due to the Syrian conflict - which increased Lebanon’s population by a quarter [7]. However, this had considerable repercussions on the country’s governance, infrastructure, economy and demography.

The health system is currently under immense strain, with its sustainability threatened due to a convergence of crises. These include an unprecedented financial and economic crisis beginning in late 2019, aggravated in the following year by the COVID-19 pandemic, and the Beirut Port explosion. Deep structural vulnerabilities have been exposed in what the World Bank has called “one of the top ten, possibly top three most severe economic collapses worldwide since the 1850s” [8]. With currency devaluation exceeding 90%, multidimensional poverty affecting 82% of households, and critical shortages of medicines and medical supplies, Lebanon’s healthcare infrastructure risks complete systems failure - reversing decades of progress on health indicators and Sustainable Development Goals (SDG) achievements.

At the heart of this collapse lies a financial system characterized by unsustainable policies and elite capture. The Ponzi-like structure of Lebanon’s banking sector, dependent on attracting new deposits to service existing debts, finally imploded in 2019 [9–11]. This was compounded by decades of corruption and fiscal mismanagement that “orchestrated by the country’s elite that has long captured the state and lived off its economic rents” [12]. In 2021, up to 82% of households in Lebanon lived in multi-dimensional poverty [13]. The resulting economic freefall has devastated all six WHO health system building blocks - from massive healthcare worker emigration to breakdowns in governance and service delivery. Patients now face impossible choices between unaffordable care or dangerous treatment delays. Health reform has been a topic of ongoing debate, yet limited progress has been made in addressing the core issues.

This study, conducted during the peak of the compounded crisis, draws on key informant perspectives to identify pathways for sustainable health system reform during Lebanon’s unprecedented institutional collapse. Through analysis of governance failures, financing models and crisis responses, we propose actionable recommendations for both immediate stabilization and long-term rebuilding. Our findings contribute to important discourse on health system resilience in fragile states. As Lebanon’s crisis represents an extreme test case for health system sustainability, the solutions explored may inform recovery efforts in other resource-constrained settings facing compound shocks.

While this study was conducted prior to the Israel-Lebanon conflict that erupted in October 2023 and escalated again in March 2026, its findings have gained even greater urgency as the war has inflicted additional damage on Lebanon’s health infrastructure. Between October 2023 and June 2025, of the 4,335 deaths due to the conflict, 241 have been health personnel, noting that there have been 163 incidents of attack on healthcare, and several hospitals ceasing to operate or only partially functioning [14]. A similar pattern is repeating again in 2026, with 2,196 killed (91 health personnel) and 7,185 wounded within a six-week period, in addition to the internal displacement of about 1.2 million Lebanese [15]. The compounding effects of military destruction, mass displacement and diversion of scarce resources to emergency response have worsened the systemic vulnerabilities identified in our research. This study’s insights into maintaining essential health services during compound crises become increasingly valuable for both immediate humanitarian response and long-term system rehabilitation.

It is relevant to highlight that the preliminary findings of this study were used to inform the National Health Strategy for Lebanon: Vision 2030, developed in 2022. The strategy process was chaired by the Ministry of Public Health (MoPH) and guided by a multi-stakeholder committee, with technical facilitation by the World Health Organization (WHO) and funding from the European Union. The authors of this paper formed the core technical team responsible for developing the strategy. This study aimed to: (1) Explore the perspectives of key persons involved in the Lebanese health system, focusing on the challenges and recovery opportunities for the short and long terms; and (2) Develop context-specific, evidence-informed health system-level policy recommendations by synthesizing these insights with lessons learned from other countries and crisis-affected settings.

## Methods

### Study design

The study design involved a qualitative approach, using semi-structured interviews with key informants related to Lebanon’s health sector. Purposive sampling was used to select key informants from the public and private sector, noting that in Lebanon the public payers are predominant, while the majority of healthcare providers are from the private sector. The proportion of private sector informants (12 of 27) reflects this service delivery landscape, where private hospitals and clinics provide the majority of curative care, and private sector stakeholders (e.g. private practitioners, pharmaceutical industry) play a substantial role in healthcare financing and delivery. As such, this composition is representative of the Lebanese health system’s public-payer, private-provider mixed model rather than a sampling limitation.

This study was guided by an interpretive research paradigm, recognizing that health system challenges are shaped by multiple stakeholder perspectives and contextual factors. A systems thinking lens informed the analysis, allowing us to examine interconnections between governance, financing, service delivery, and workforce dimensions. Key informants were selected based on their expertise in public health, hospital management, healthcare financing, and policy-making. Selection was criterion-based, prioritizing informants with:

- Decision-making authority in health system governance.
- Frontline implementation experience.
- Sector-specific expertise across the health system landscape.

We explicitly sought to represent institutional hierarchies (policymakers to practitioners) and health system functions (financing, service delivery, regulation). We chose open-ended interviews rather than other tools such as focus group discussions, as interviews would allow more individualized and detailed responses, as well as allowing informants to not be restrained by group dynamics and be more at ease in discussing sensitive topics such as political interference and accountability. In addition, given the specific expertise of key informants, some may be more capable to share in-depth insight on select topics in an individual rather than group setting.

### Interview guide

An interview guide was developed and pilot-tested, and following modifications for clarity and intelligibility, this was revised into an interview guide with a total of 35 questions (see supplementary file). This was undertaken as part of the Lebanese National Health Strategy development. This combined closed-ended questions and open-ended probes, designed to elicit detailed responses regarding the current challenges in the healthcare system and potential reforms needed to improve health service delivery and outcomes.

The interview guide questions covered topics such as:

- The role of public and private healthcare providers.
- Health system governance and accountability mechanisms (or dysfunctions).
- Financing mechanisms and the potential for reform.
- Human resources and the retention of healthcare professionals.
- The impact of the COVID-19 pandemic on health sector resilience.
- Approaches to improve access to affordable and equitable healthcare.

### Key informants

A total of 27 key informants were interviewed (eight women, nineteen men), ensuring diverse perspectives from both the public and private sectors. This included persons with a background in public institutions, hospitals, primary care centers, nurses, physicians, pharmacists, universities, non-governmental organizations, private insurance and the pharmaceutical sector (see Table 1). Individuals referred to as “leaders” in Table 1 were identified based on formal institutional positions (e.g. directors of hospitals or primary care centers, heads of syndicates or orders, leaders of insurance funds) or recognized expertise and decision-making authority within their respective fields, as determined through the criterion-based sampling approach described above.

### Data collection

The interviews were conducted between March and May 2022, using virtual meetings due to COVID-19 pandemic considerations. Interviews were conducted in either Arabic or English language, based on the participant’s preference, and were audio recorded and transcribed with participant consent. All the interviewers (JK, HS, AH, HH) had several years’ experience in health research, and educational backgrounds with an MPH and/or MD degree. Both HH and JK have more than 15 years’ experience working at or with the MoPH. Two researchers participated in each interview, one being the designated interviewer, and the second being note-taker. Each interview lasted approximately 60 to 75 min.

### Data analysis

Data were analyzed using thematic content analysis, guided by the approach specified by Braun and Clarke (2004) [16]. Thematic analysis was selected over content analysis because our objective was to identify and interpret patterns of meaning across the data through an inductive, iterative process of theme development. While content analysis can examine both manifest and latent content, it typically relies on coding based on predetermined categories, whereas thematic analysis offered greater flexibility to capture emergent patterns and the complexity of informants’ perspectives without being constrained by a priori frameworks. To familiarize themselves with the content shared by the key informants, the researchers (JK, HS, AH) carefully reviewed all transcripts multiple times before initiating the coding process. This helped them grasp the scope and nuances of the responses received. Each statement was preserved in its original form and assigned descriptive codes. Nvivo 12.0 software was used to support the coding procedure. These coded statements were then examined in connection with the study’s guiding research questions. The preliminary themes were further refined based on the analysis of the data. Similar codes were grouped to form subthemes. Illustrative quotes were extracted to highlight the perspectives of informants.

### Reflexivity statement

The research team included individuals with longstanding engagement in the Lebanese health system. Three authors (JK, HH, RH) have more than 15 years of experience working with the Ministry of Public Health (MoPH), and one author (HH) was a current MoPH staff. Two authors (AR, RH) are affiliated with the WHO Country Office for Lebanon. One author (WA) is a former Director-General of the MoPH with over 25 years of experience; at the time of the study, he was retired from the MoPH and independently contracted by WHO as a consultant for this work. To manage potential biases arising from these relationships, several approaches were employed. First, the interview guide was developed collaboratively and pilot-tested with an independent researcher (Prof. Michèle Kosremelli Asmar) not involved in health system governance. Second, interviews were conducted by pairs of researchers, with all interviewers having no direct supervisory relationship with the informant. Third, data analysis was led by an author (JK) with prior MoPH experience but no current operational role, and coding was reviewed by team members with diverse institutional perspectives (AH, HS) to challenge assumptions. Fourth, preliminary findings were shared with informants through subsequent online discussions and presentations, providing an opportunity for validation and refinement of interpretations. Finally, the inclusion of direct quotes throughout the Results section allows readers to assess the alignment between interpretations and informant testimony. This study adheres to the Consolidated Criteria for Reporting Qualitative Research (COREQ). The completed COREQ checklist is provided as Supplementary File 1.

**Table 1:** a and b: Description and sector/institution of key informants

**a) Description of informant**	**Number of informants**
Finance expert	1
Health professional	1
Health researcher	3
Health services leader, public authority	1
Health services leader, physician	1
Insurance fund leader, public fund	2
Insurance leader, private insurance	2
Journalist	1
Major hospital leader	2
Member of Parliament	2
Nursing leader	2
Patient association leader	1
Pharmaceuticals industry	4
Pharmacists leader	1
Physician leader, private sector	1
Primary care center leader	1
Syndicate leader	1
**Total**	**27**
**b) Sector/institution of informant**	**Number of informants**
Academia	3
NGO	1
Order of Nurses	1
Order of Pharmacists	1
Politician	2
Private sector	12
Public sector	5
Syndicate of Private Hospitals	1
UN Agency	1
**Total**	**27**

## Results

The analysis of the interviews identified six key themes regarding Lebanon’s health system: healthcare governance and accountability, health system reforms and financing, healthcare access and delivery, health personnel, health information systems, and people-centered health system. Each theme included several subthemes, reflecting both manifest content (explicit) and latent (implicit) content, highlighting the breadth and depth of challenges facing the system. The themes and subthemes are listed in Table 2, alongside illustrative quotes.

**Table 2:** Themes, subthemes and illustrative quotes

Theme	Subtheme	Illustrative Quotes
1. Healthcare Governance and Accountability	Accountability Deficit	“When I know that there is an official who smuggled medicines […] and sold them in the July war, I would like to see him in prison, and not free after only two days because of a phone call […]” (P11)
	Needing Leadership and Strategy	“The health strategy should not be owned by the minister alone; it should be owned by the government […] Every minister should implement the part that he reaches and the other continues.” (P21)
	Political Interference and Corruption	“Political interference always seeks the special needs of special populations: their special populations.” (P15)
	Mixed Views on Public-Private Partnership	“At the end what matters is that the public does the two things it has to do. The first is to have the services that cater for the most fragile part of the population, and the second is to organize the system in a way that is optimal for the private institutions.” (P16)
	Mismatched Role of the Ministry of Public Health	“In financing of health, the situation in which the Ministry of Health is the only ministry that spends money without even knowing that it has the money to spend must stop immediately.” (P16)
	Potential of Lebanon as a Regional Hub	“If I were in a position in the government I would say yes: it is a plus for the country. Is it realistic today? I don’t think so, with the brain drain.” (P10)
	Emergency Preparedness and Health Security	“[Globally and in Lebanon] the focus was on the hospitals and they forgot the important player which is the primary healthcare. Primary healthcare is within communities, so the work should have started [there].” (P24)
2. Health System Reforms and Financing	Major Reforms or Weathering the Crisis	“This is the chance. I think this is an opportunity, if everyone is reviewing everything, if we have a strong speaker with a simple plan. This is the time to say for example: ‘Hello, we will have one insurance system, no more ministry and this is the time to implement [unification of funds].’” (P3)
	Rethinking Financing	“We were dealing with health as if we were in a rich country, while we know that we have become one of the poorest countries in the world […] we must work out a new policy and all the Lebanese to be convinced that we have become a poor country.” (P23)
	Unification vs. Harmonization of Public Payers	“Unifying the public funds will decrease the government’s overhead, it will facilitate the implementation of electronic medical files and the standardized medical card later on.” (P2)
	Public Sector Strengthening	“At the end, you are building institutions, and these institutions are built by those who are in charge. So in fact, what we need is to have very clear, prerogatives being given, powers being given, to those who are managing the hospitals and at the same time, very clear checks and balances at the high level to make sure that if people don’t perform there, they just don’t stay forever.” (P16)
	Private Sector and the Common Good	“I think the issue of proper control over the private sector is a critical one. It doesn’t mean that they have to be over-burdened, but there is a minimum required that is not there.” (P16)
3. Healthcare Access and Delivery	Role and Strengthening of Public Hospitals	“We should restructure all the public hospitals […] We should reconsider everything even our mindset towards public hospitals, the way the government treats them, the way people get hired in them.” (P2)
	Role of Private Hospitals	“The government needs to pay its dues yes, but not financial support, this is nonsense […] When the Central Bank Governor says to the private companies ‘I will subsidize’ […] he needs to keep his promises.” (P1)
	Package of Services	“This is one of the very few safety nets that remain […] I would keep the generous package, but I would strengthen very much the controls over what is happening.” (P16)
	Affordable and Rational Tertiary Access	“The main essence of insurance coverage is no longer possible, because you cannot expect what would be your expenses, unless you price it in fresh dollars (newly earned hard currency).” (P7)
	Developing a National Emergency Service	“Since thirty years they wanted to implement SAMU in Lebanon [French integrated emergency medical service]. The Red Cross is available; how can you make SAMU work in Lebanon.” (P3)
	Collaboration for Primary Care	“The simple fact that public authorities have to rely on NGOs mean that there is something wrong in the system […] There is a lot of cleaning to be done in the NGO environment and there is a lot of rationality that needs to be introduced in the approach of the Ministry.” (P16)
	Medications Access, Sustainability, and Central Bank Role	“Now the problem of accessibility and availability is only due to no transparency, stupidity and the pretention that the Minister knows everything and the Central Bank knows what to do.” (P1)
4. Healthcare Personnel	Nurses Emigration and Working Conditions	“[We] have to ‘live’ in order to live in a shortage. Nurses are not ‘living’ because their income is so low. Very simple, how can you take care of others, if you are not able to take care of yourself.” (P22)
	Physicians Emigration and Working Conditions	“The best physician’s fees today are 10$, and the less experienced physician’s fees are much lower. This is abnormal and needs solving.” (P2)
	Strengthening Primary Care in Rural Areas	“If there is a school in Hermel and you ask the teacher you want to live in Beirut or Hermel, he will say Beirut. Why? Because I will have better life, and a better salary, and I can send my kids to a better school.” (P3)
5. Health Information Systems	Electronic Health/Medical Records	“You cannot access the EMR of each hospital, but you can access EHR that will give you the essential information needed, especially the emergency rooms to minimize duplication.” (P9)
	Information Infrastructure and Development Opportunities	“It has to be done at the lowest level for a start, it has to start from pharmacies and practitioners and upload it to a national data center that is performant.” (P16)
6. People-Centered Health System	Robust Gatekeeping	“[We] have to make sure that the gatekeepers or the family physicians or the centers or others receiving the patients are competent […] Otherwise, such a system cannot be sustained.” (P20)
	People-Centered Care	“There should be a patient-representative body […] Even when we meet there isn’t a patient representative at all healthcare institutions, and we cannot be in the shoes of the patient.” (P8)

### Healthcare governance and accountability

#### Accountability deficit

Several informants identified the need for a ‘culture’ of accountability, with governance mechanisms in Lebanon’s health system being described as weak and susceptible to political interference, of which sectarian clientelism is a main driver. The judiciary’s slow response to case referrals and limited enforcement options for public payers were attributed by informants to sectarian patronage networks that prioritize communal loyalty over institutional accountability. A major barrier to accountability is the affiliation of politicians with particular health institutions and officials.

When I know that there is an official who smuggled medicines […] and sold them in the July war, I would like to see him in prison, and not free after only two days because of a phone call […] Every time we try to get close to one of the thieves, his sect comes and defends him from the top of the pyramid to the bottom of the base. They do not understand that corruption has no religion or color. (P11)

Informants suggested strengthening the inspection and audit functions of the State; technocratic insulation by appointing/empowering independent health experts who are ‘free from politics’; and using key performance indicators, accreditation and benchmarking. Informants also suggested the creation of a higher level of governance, transcending institutional fragmentation: *“If we do a National Health Council*,* it will have access to all of the data*,* and we will know who is doing what.” (P7).*

#### Needing leadership and strategy

Informants considered that having a clear vision and strategy is critical for the health sector but are generally missing, and these should be communicated to the public. They suggested a complete transformation in how budgets are set, how we think of health, and not limit change to regulation or legislation alone.

Issues related to changing policies with changing ministers were highlighted, which interrupt the development of the health sector; *“We had a minister and he left; the project also left.” (P17)*. Informants suggested that the entire government should have ownership of a health strategy, and not solely the health minister. This would improve accountability, reduce reliance on individual health ministers, and ensure continuity across successive government – which is a critical consideration in Lebanon’s volatile political context.

The health strategy should not be owned by the minister alone; it should be owned by the government […]. Every minister should implement the part that he reaches and the other continues, the work should be cumulative, and this is how policies should be. (P21)

Informants also noted that health should be more highly prioritized by the State, otherwise this would also threaten social stability. Informants proposed that stakeholder engagement on health strategy should be an ongoing activity, rather than a one-time activity.

Some informants noted the need to break away from silo thinking and towards system-building and institutionalization, with more effective collaboration among stakeholders, including the MoPH and international agencies. Pervasive clientelism needs to be addressed, and current capacities should be used in a more effective manner that prevents profiteering.

The essential basic thing is to have a clear health policy of where to go, what we want, and what is needed for this […]. There is clientelism at all levels, and there are individuals and not a system. When there is no system, you cannot build. When one person goes, everything goes. There are individual attempts and good intentions, but the rest is not present. (P24)

There was also concern that unless there is a nationally developed approach for the health sector, then any major reform would be driven by the interests of major external funders (e.g. International Monetary Fund) rather than national interests. A national voice would be able to engage different entities and harness support in the interests of the public. Some informants noted that politicians in key positions on parliamentary committees or ministries are capable to block new legislation and initiatives, sometimes due to pressure from their political parties.

[A national voice would say: ] ‘this is what we feel we can do and we want to do; you want to support us, you are welcome, but this is direction we want to go.’ (P20).

#### Political interference and corruption

Political influence on healthcare leadership emerged as a significant barrier to reform. Informants noted that it is challenging to protect against political interference, as the health sector is service-based. Health services are considered a channel for politicians’ generosity, rather than as part of people’s rights, an example of clientelism in practice. Political parties consider ministries to be ‘their share’, and politicians act in their own self-interest: *“Political interference always seeks the special needs of special populations*: *their* *special populations.” (P15)*.

Most informants suggested countering interference through creating independent organizations, strengthening the public administration, and improving the process of appointing civil servants. Independent bodies increase the distance between politicians and people seeking services, such that politicians cannot function as ‘go-betweens’. Examples noted as requiring reform included public hospital boards (appointed by the Council of Ministers) and some primary health centers. Developing mechanisms for selecting the best candidates for specific roles and presenting politicians with a short-list of highly qualified persons would also be an approach to limit interference (i.e. merit-based recruitment). Other approaches suggested by informants were increased reliance on data and standardized service packages. Some informants noted that interference would decrease once the health sector meets the needs of the majority of the population.

I think I have worked with 30 ministers, as soon as the minister comes, he thinks he is the only one to understand what is to be done. […] He is here to motivate his ministry, to take the best available resources of the ministry to build on all the assets that have been built in the ministry before he came […] We have to build strong Public Service. This was the situation at the time of President Chehab [1958–1964]. When somebody was the director-general of a ministry, it was the best that the Lebanese could obtain. They were the best paid and the most respected. Now it is the opposite. (P1)

Informants emphasized the need for an effective Central Inspection and an independent judiciary, highlighting that reducing political interference requires both legal foundations and an independent judiciary. Some also suggested private companies manage public hospitals to distance politicians from service delivery, improving efficiency and accountability.

Let me put it very bluntly: we have a system that is not good. But even under this system, if you choose the right people, those who are willing and able and have the capacity of stopping interference in their sector because they are professionals, because they do their job properly, it is always better than to choose anybody who doesn’t […]. So, in this system whereby we have heads of clans who are sitting outside the government, nominating, appointing people just to serve them; it is difficult. But if we come back to a system whereby big figures have their reputation, are trustworthy and strong, then its already a big improvement. (P16)

Informants underscored the need to guard the health sector from politicians. Countering corruption and interference is difficult in the current context and needs a long-term effort, but a start is needed today, as it is a priority. Some informants shared their own experiences regarding political interference and its impact, such as the recent medication crisis, which left many cancer patients without treatment and led to street protests by some patients and their families.

They kept sending us back and forth for three months between the Central Bank and the health ministry […] So we had to go protest on the streets for them to start taking small measures. (PX)

#### Mixed views on public-private partnership

Views on the effectiveness of Public-Private Partnerships (PPPs) in the health sector were mixed, with some informants noting mistrust and lack of cooperation. Informants suggested that non-financial incentives may have a role in encouraging PPP, including training certifications and research initiatives. They also highlighted the importance of strengthening the public sector, so any partnership with the private sector would be an equitable and effective one, and also avoid profiteering. Informants noted that PPP should be considered as a tool, but not as a solution itself or as being intrinsically good. It is more important to address root causes, rather than ignore them and use tools such as PPP.

At the end what matters is that the public does the two things it has to do. The first is to have the services that cater for the most fragile part of the population, and the second is to organize the system in a way that is optimal for the private institutions […] I don’t believe that when this doesn’t work, then complicated PPPs will work. One has to start with the fundamentals […]. (P16)

The absence of an independent judiciary was considered by informants to be a major barrier to having effective PPPs, as well as political interference. Proposed measures included appointing private-sector representatives to public hospital boards or outsourcing hospital management to private-sector professionals.

#### Mismatched role of the ministry of public health

The Ministry is regarded as a key player in driving governance reforms but lacks both independence and resources needed for effective oversight. Informants noted that the MoPH is diverted from its primary regulatory role due to its significant involvement as a public payer. Instead, the Ministry should focus on developing the national health strategy and overseeing the health sector, ensuring good governance, efficiency and accountability. This includes leading efforts on personnel development, developing the national health information system, contingency planning and formulating health policies. Some informants proposed creating a separate body to handle implementation and technical details, such as health technology assessment and accreditation, similar to France’s Haute Autorité de Santé (HAS). Others suggested establishing a higher health authority, like that in the UAE. The current crisis has exacerbated this role mismatch, as rising unemployment has led to a shift in coverage from the National Social Security Fund (NSSF) to the MoPH.

The simple fact that the Ministry becomes a service provider […] is preventing it from preforming the basics that it should perform.” (P16).

Some informants considered that the Lebanese context presents unique challenges that hinder the health ministry from functioning as it does in other countries. These challenges include the pervasive roles of sectarian clientelism and corruption.

Unfortunately, in Lebanon every entity has a political and sectarian affiliation and wants to own its separate fund to benefit from and provide services from. (P4)

#### Mixed views on Lebanon’s future potential as a regional hub

Some informants suggested Lebanon’s potential role in regaining its reputation as a regional hub for healthcare services, given its geographic location and historical expertise in medical education. However, they argued that this would only be possible with significant improvements in governance and infrastructure, and that it was neither realistic nor feasible, at least for the short term and until the current crisis resolves. Another suggestion was that a regional hub may be pursued for a specific rather than a wide range of services.

If I were in a position in the government I would say yes: it is a plus for the country. Is it realistic today? I don’t think so, with the brain drain. Is it feasible? It depends on what we want in our system and how we are defining it, and how are we designing it. (P10)

Informants opposed to pursuing a regional hub status argued that the health system should instead focus on rebuilding services for the local population. Some informants noted that the region has moved on in the past few decades, and other countries now have advanced health services and a stable context, such as Qatar and the United Arab Emirates. They noted that Lebanon has largely lost its historical advantage of its extensive experience and human resources due to the crisis and emigration of health professionals.

I think that to continue to look backward all the time is not a good thing. Lebanon should be looking at the future and at how much the future is going to be different, not continuing in this nostalgia issue. (P16)

The need to attract hard currency was identified as the main reason for offering health services to foreigners. However, any push to boost medical tourism should prioritize improving Lebanon’s health personnel. Other suggestions included allowing hospitals to implement dual pricing: discounted rates for Lebanese and higher rates for those coming from abroad. Informants also noted the need to invest in the development of people, their mindset, work ethic and cooperation, which has been a neglected priority.

#### Emergency preparedness and health security

Informants stressed the need to invest in disaster preparedness and consider various scenarios. They highlighted challenges with collective work in Lebanon. Some also pointed out that the primary care sector was largely excluded from the COVID-19 response. Some informants considered that the national pandemic response lacked coordination and accountability. They stressed the need for a single spokesperson for health crises, providing regular updates, while also involving external professionals who can speak freely and offer critical perspectives.

[Globally and in Lebanon] the focus was on the hospitals and they forgot the important player which is the primary healthcare. Primary healthcare is within communities, so the work should have started [there]. (P24)

### Health system reforms and financing

#### Major reforms or weathering the crisis

Informants expressed divergent views on whether the current crisis warrants immediate structural reform or prioritizes stabilization. This tension between short-term crisis management and long-term transformation permeated discussions across all themes. Many informants believe now is the right time for major changes in the health sector, while others argue the political and economic context is unfavorable. Some see the current period as a transitional phase, where new legislation is hard to implement but a vision and strategy should be developed for post-election reforms. During this transition, the focus should be on crisis management while preparing for broader reforms. Some informants noted major change is necessary in the short-term, as the ongoing crisis’ impact cannot be sufficiently mitigated by minimal changes.

This is the chance. I think this is an opportunity, if everyone is reviewing everything, if we have a strong speaker with a simple plan. This is the time to say for example: ‘Hello, we will have one insurance system[…] this is the time to implement [unification of public payers of healthcare].’ (P3)

#### Rethinking financing

Informants noted that available financing was limited and insufficient to meet population needs. They called for a rethink of financing, including unifying or harmonizing public funds, and increasing transparency. Some supported keeping the NSSF contributions even under major reforms, so as not to lose this as a financing source.

Some informants argued that Lebanon cannot fund health services through higher NSSF contributions or increased taxes, as revenues are already low and higher taxes would raise the cost of living. They also cautioned against asking for higher co-payments from the public, as many have lost savings, have limited income, and face a dire economic situation.

We were dealing with health as if we were in a rich country, while we know that we have become one of the poorest countries in the world […] we must work out a new policy and all the Lebanese to be convinced that we have become a poor country. (P23)

Informants called for international financial support during the transition, with sustainable health financing depending on national economic recovery. They suggested increased taxes on tobacco, alcohol, and similar products to boost revenue. Given Lebanon’s current vulnerability, they stressed that the state should prioritize health.

#### Unification vs. Harmonization of public payers

Most informants supported unifying public payers. In the short-term, however, some viewed it as unrealistic, to the crisis and the extensive preparations required. Harmonization was seen as a transitional step toward eventual unification. Informants highlighted the benefits of unification, including increased efficiency, reduced overhead, simpler system management, and a stronger position for negotiating with providers and suppliers. Unification would also allow for easier implementation of other initiatives and improve healthcare equity.

Unifying the public funds will decrease the government’s overhead, it will facilitate the implementation of electronic medical files and [and other reforms] later on; and it will facilitate and change the purchasing system where all purchasing will become unified and promote the volume rebate […] the payer in all cases is the Lebanese government, so it does not make sense to pay through all these entities, which is of course rooted from political interests. (P2)

If I want to be biased to the institution I represent […] I would say keep them separate as they are […]. But I will not proceed from this […] All Lebanese should be under one healthcare that has the same laws and regulations and provides the same services based on the principle of equality among citizens. (PX)

Some informants viewed harmonization as more realistic due to political interests in maintaining separate payers, which they saw as the main barrier to unification. However, harmonization may still be difficult to achieve because of differing financial capabilities among payers and the history of challenging collaborations, such as between the MoPH and NSSF.

We shouldn’t have all these fragmented entities. If it is inevitable that they exist, they should be working in one way [i.e. harmonized]. I think this causes a lot of confusion in the hospitals, an additional cost and inequality. For example, those covered by Army don’t pay out of pocket, those covered by the Ministry pay 15%, those covered by NSSF pay 10%. In all this chaos, the Ministry lost its role to all these branches of public insurers. (P14)

Suggestions related to harmonization and gradual progress toward unification included unified procurement, personnel, and training, as well as a single electronic platform. It was also proposed that a higher health authority supervise the separate funds in the context of harmonization. A third alternative was to have a third party manage all public payer functions. Additionally, increasing the presence of mutual funds was recommended to reduce the financial burden on patients.

Many informants also emphasized that Lebanon’s healthcare system is overly dependent on external financial aid, especially for public hospitals, making the system vulnerable to fluctuations in international assistance.

#### Public sector strengthening

Strengthening the public sector in healthcare was seen as crucial for long-term sustainability. Informants cited political interference, corruption, and lack of accountability as key factors in its decline. While the ongoing crisis complicates reforms, the rising cost of private sector services is driving more people to public healthcare, creating an opportunity to rebuild the sector.

Some informants suggested that strengthening the public sector requires investing in personnel quality, including recruiting competent staff, establishing clear role descriptions, conducting performance appraisals, and implementing accountability mechanisms.

At the end, you are building institutions, and these institutions are built by those who are in charge. So in fact, what we need is to have very clear, prerogatives being given, powers being given, to those who are managing the hospitals and at the same time, very clear checks and balances at the high level to make sure that if people don’t perform there, they just don’t stay forever. (P16)

Suggestions included equipping public facilities, securing short-term external support, exploring funding models, and investing in digitization. Informants also recommended greater collaboration among public hospitals, primary care centers, and increased community engagement.

#### Private sector and the common good

Informants viewed a strong private sector as beneficial if properly regulated, which is not currently the case. They stressed that the public should define the common good, with both sectors working toward it, free from private sector profit influences. The private sector should be viewed as a collection of distinct entities—health professionals, hospitals, and pharmaceuticals—each requiring tailored engagement. Effective leadership and regulation are essential to prevent exploitation of the common good.

I think the issue of proper control over the private sector is a critical one. It doesn’t mean that they have to be over-burdened, but there is a minimum required that is not there. (P16)

Some informants felt the private sector relies too heavily on government contracts, while others saw it as neglected due to mismanagement. This requires rebuilding trust and collaboration between the two sectors to address the crisis.

### Healthcare access and delivery

#### Role and strengthening of public hospitals

Informants recognized the crucial role of public hospitals but highlighted issues with resources, equipment, and management. There is considerable variation in service quality across facilities, with lower-performing hospitals adversely affecting both health outcomes and system efficiency. They stressed the need for clearer accountability and improved transparency and governance.

We should restructure all the public hospitals [outside the crisis]. We should reconsider everything even our mindset towards public hospitals, the way the government treats them, the way people get hired in them, their financial independence, and increasing their accountability. (P2)

Informants stressed the need to define the roles and relationship between public and private hospitals. They questioned whether the State should aim for most hospitalizations in the public sector (as in France), noting that private hospitals are essential due to the shortcomings of public hospitals. While local community-based public hospital boards are generally beneficial, they may not be effective in Lebanon due to corruption and interference. Aligning individual and institutional interests could improve hospital management and healthcare delivery.

Theoretically, in a normal less corrupt environment, this arrangement would work. With a more corrupt environment and threats to discredit the public hospital, they would need other forms of protection. (P3)

Informants believed public hospitals should be central to crisis response, but most were crucial yet not indispensable during COVID-19 and absent in the Beirut port explosion response. Hospitals relied on external donor financing, making it difficult to assess their cost-effectiveness. The crises showed that public hospitals could play a key role if properly resourced. Informants were disappointed by the weak involvement of private hospitals in the pandemic, seeing it as further justification for increased investment in public hospitals, and clarifying the role of public and private hospitals.

I think public hospitals should be the role model of hospitals. (P14)

What went bad in the crisis that all the strategy of fighting COVID was laid on the public sector for more than one year and it was not very effective, and there was a lot of pressure on the private sector, only some public hospitals were able to have an acceptable response, many others did not. The private sector was not ready or was not willing […]. (P7)

Most informants disagreed that public hospitals should focus only on the low-income population. Both public and private hospitals are needed to increase access for the economically vulnerable, with public hospitals also serving the non-poor, as most cannot afford private care. Some informants warned that linking public hospitals only to the low-income population could increase inequities and lower perceptions of care quality. They also stressed that private hospitals share the responsibility to care for the vulnerable.

#### Role of private hospitals

Most informants opposed using public funds to support at-risk private hospitals, seeing it as cost-ineffective. Instead, they suggested reimbursing overdue payments to hospitals, and improving reimbursement tiers. Some informants noted Lebanon’s oversupply of hospitals, particularly private ones, suggesting closures could benefit the system. However, closures would be politically unpopular unless public hospitals could fill the gap. Mergers and indirect support, such as solar panels, were suggested for hospitals in critical locations to maintain local healthcare access.

The government needs to pay its dues yes, but not financial support, this is nonsense […]. When the Central Bank Governor says to the private companies ‘I will subsidize’ […] he needs to keep his promises. (P1)

#### Package of services

Some informants suggested revising the healthcare services package, starting with developing protocols for efficiency, prioritizing services, and reducing them if necessary, while others suggested improved allocation and funds of the ministry rather than adjusting the service package. Most informants agreed that primary care, hospital care, and chronic care services (including medication) should remain included, as they are vital for vulnerable populations and promote equity.

This is one of the very few safety nets that remain […] I would keep the generous package, but I would strengthen very much the controls over what is happening. (P16)

The need to monitor health outcomes and conduct cost-effectiveness analyses was noted, with the Lebanese Drug Authority handling medication assessments (currently not implemented). Some of the newest, most expensive medications and prostheses were suggested for exclusion.

Other suggestions included varying medication subsidies, empowering the MoPH medications committee, and training physicians to better understand the health system and their role in safeguarding it. Informants suggested considering coverage eligibility based on the condition, rather than the individual, and emphasized the need to identify all covered individuals, not just service beneficiaries. They also highlighted the need to clarify expatriate coverage.

#### Affordable and rational tertiary access

Informants noted reduced affordability as the main reason for decreased hospital access, along with supply disruptions, lack of devices, the COVID-19 pandemic, and a decline in the number and quality of health personnel. Informants highlighted widespread impoverishment, with most people unable to access their bank savings and/or not earning sufficient hard currency (popularly known as ‘fresh’ dollars). Since hospitals buy supplies with hard currency, hospital care has become unaffordable. While public payers like the NSSF still approve hospitalizations, they use a much lower exchange rate, effectively reimbursing only 10% of the bill rather than 90%, leaving patients to cover the difference. Private insurers and third-party payers face similar challenges.

The main essence of insurance coverage is no longer possible, because you cannot expect what would be your expenses, unless you price it in fresh dollars (newly earned hard currency). (P7)

The cheapest hip prosthesis costs 2,800-3,000 dollars, which is the salary of two years for the average Lebanese employee. (P2)

Informants reported that people have decreased their visits to hospitals, due to financial barriers. This results in increased chronic disease complications, delayed hospital presentation, and increased mortality.

There is a big topic that is not given much focus, which is the issue of the increasing death rate in hospitals because people do not go to the hospital as quickly as necessary, especially patients with heart diseases and blood pressure diseases, and so on, due to the lack of money, so they arrive at the hospital in a late state. (P23)

#### Developing a national emergency service

Informants valued the Lebanese Red Cross, Civil Defense, and nearby hospitals’ responses after the Beirut port explosion, but noted insufficient capacity. They emphasized the need for a central organization to coordinate between first responders and hospitals, and to direct patients based on availability. Such a central body would answer patients who ask *“This is my case. Where do I go? What do I do?” (P17)*. The dilemma of where to strengthen the emergency care chain was also noted by some informants.

Since thirty years they wanted to implement SAMU in Lebanon [French integrated emergency medical service]. The Red Cross is available; how can you make SAMU work in Lebanon. If you made Red Cross to be advanced, the ER then will not be advanced enough. If you want to make a regional hospital with a regional advanced emergency, then you’ll get opposition why it is not for a certain sect in Lebanon. Or you cannot reach it because of bad roads. There are a lot of challenges in this. Politics, payment system and compensation of experienced providers in emergency services. (P3)

Informants noted the major financial barriers, as well as road conditions, equipment shortages, and political factors as obstacles to care. They also noted that refugees are better covered for emergency care (MedNet) than most Lebanese citizens, who lack coverage (e.g., MoPH, NSSF), and the fragility of health systems when faced with crises such as pandemics.

Emergency cases problem was especially discovered with COVID-19 when cases increased, and we did not have a proper planning for that. And maybe in Europe they also didn’t prepare for such high numbers of emergency cases. This reflects the fragility of the health system in a country. (P6)

#### Collaboration for primary care

Informants supported continuing the successful collaborative approach to the national primary care network, partly attributed to community-minded primary care leaders. Some also noted the expansion of centers due to the Syrian refugee influx. Informants noted variation in service quality, recommending standardization, capacity building, and investment in personnel, maintenance, and user fees. They also pointed out areas lacking or with poor-quality centers. Some informants questioned long-term sustainability due to reliance on NGOs, noting over-dependence on external aid especially for international NGOs, and political/sectarian affiliations. They suggested linking centers more closely with local municipalities rather than other organizations.

The simple fact that public authorities have to rely on NGOs mean that there is something wrong in the system […] There is a lot of cleaning to be done in the NGO environment and there is a lot of rationality that needs to be introduced in the approach of the Ministry. (P16)

Informants stressed that strong leadership and governance are key to building public trust in primary care. Regular evaluations would improve services and transparency. Some warned the network may collapse due to the MoPH’s inability to maintain resources, relying instead on aid and donations. Informants raised concerns about transparency, especially that financial reporting is made to funders but not the State. Public access to this information would support sector development, as the cost-effectiveness of centers remains unclear due to financial opacity.

They [other countries] are sending funds because Lebanon collapsed, as is done with the governments that cannot take care of their citizens, or with the third world countries. (P19)

#### Medications access, sustainability, and central bank role

Informants considered the then-Central Bank governor to be primarily responsible for the medication crisis due to his failure to allocate promised funds for subsidies, causing importers to delay supplies. Some also deemed the long-standing USD-LBP peg a catastrophic decision.

Now the problem of accessibility and availability is only due to no transparency, stupidity and the pretention that the Minister knows everything and the Central Bank knows what to do [instead of engaging stakeholders…]. When promises are given by a public officer, they should be fulfilled. (P1)

Informants recommended prioritizing subsidies for life-saving medications, using local manufacturers for chronic disease drugs, diversifying supplies, ensuring quality control, and adjusting pricing based on purchasing power. All of this requires strong political leadership, which is absent.

We are living in chaos. A lot of people cannot be on their medications. And they are reaching hospitals at last stages of their disease. This government has impoverished its people. The best way people can buy their medications is that we change politicians. (P14)

Informants proposed tackling medication smuggling out of Lebanon, adopting electronic prescriptions, directly subsidizing individuals (rather than medications), and having primary care centers distribute medications with minimal or no co-payment. Some informants noted that the pharmaceutical industry sees the State as unwilling to address the medication crisis. They also suggested reducing the gap between imported and local medications (1.5 billion vs. 100 million units) to lower costs while maintaining quality. Informants emphasized cost-effectiveness analyses to avoid expensive medications with limited benefits, suggested delaying new drug adoption to cut costs, and prioritizing essential medications. Some, like chemotherapy, must remain subsidized due to their unaffordability.

What strikes me […] is that usually the people who are in the most difficult situation are faced with the most difficult access to medication. (P16)

Across these subthemes, a consistent pattern emerged: while the infrastructure of healthcare access and delivery remains nominally intact, its functionality has been severely compromised by financing disruptions, governance failures, and workforce attrition. Each of these reinforcing the others in a downward spiral.

### Healthcare personnel

Informants were explicit that the main factor threatening human resources is the financial compensation, specifically since the onset of the crisis in 2019. This applied to all persons working in the health field public and private sectors, as well as to all other occupations.

The physiological need of the employees in all sectors including the public sector, which is the base of the pyramid, is not present […]The minimum should be maintained, we reached a place where we need to maintain their daily need of food. (P5)

Today, any payment given to nurses, pharmacists or doctors cannot secure the necessities of life […] Everyone wants to stay in Lebanon, but the issue is money. (P6)

Some informants noted that this particular crisis is eroding the national capacity of health personnel far more that previous crises throughout the past century. Reversing this was expected to be very challenging.

#### Nurses emigration and working conditions

In spite of their importance, informants considered nurses in Lebanon to be highly under-paid, and to such an extent that their own livelihood and well-being is threatened. Salary was not a major factor for retention prior to the crisis, but it is now. Informants suggested that some nurses have remained in Lebanon because they are either being fully or partially paid by ‘fresh’ dollars (newly earned hard currency), particularly by some NGOs. However, most hospitals and public payers do not have access to such currency. Concurrently, nurses are offered work abroad which secures their family’s well-being. This inevitably impacts health system capacity. Informants suggested incentivizing nurses through greater recognition of their role as equals to doctors, more opportunities for growth, leadership positions in primary care centers, and improved working conditions, though these are not a solution to the main challenge of finances.

[We] have to “live” in order to live in a shortage. Nurses are not “living” because their income is so low. Very simple, how can you take care of others, if you are not able to take care of yourself. (P22)

I know several hospitals who had to close their department because there are no nurses, so currently the only incentive is money. (P8)

#### Physicians emigration and working conditions

Informants identified financial compensation as a key factor in doctor retention. Public and third-party payments are low, and physicians cannot raise consultation fees due to decreased affordability. Older doctors are likely to stay in Lebanon, but many middle and senior physicians have emigrated, leaving departments with less experienced leadership, which affects the quality of medical education. Informants suggested third-party monthly payments for physicians and hospitals, allowing physicians to split their work between Lebanon and nearby countries, increased use of telemedicine, and paying part of physicians’ pensions in US dollars. They emphasized that a recovery plan involving public payers, hospitals, physicians, and other professionals is essential due to the interdependence of financial compensation.

The best physician’s fees today are 10$, and the less experienced physician’s fees are much lower. This is abnormal and needs solving. (P2)

It is a chain, and to limit that, that’s a question of -again- good governance at the level of the whole country. We are becoming a laughing stock, there is no other word. (P16)

Some participants noted that physician emigration has long been the norm in Lebanon, and the current trend is not unusual, reflecting the need for long-term planning of the health workforce, including qualifications and roles.

If you have 6 medical faculties you will have 600 physicians per year for a country that needs 60 physicians per year. (P3)

Whether physicians work individually or as part of a larger group was also noted. The current crisis may be an opportunity to reconsider Lebanon’s physician employment model, as most physicians are not salaried, and alternative models could be explored.

Every physician had his own silo and defending his own silo. There are a lot of models, the world had discovered and is being implemented and working and have references, we did not even think about it yet. We need to change the processes. (P9)

#### Strengthening primary care in rural areas

Informants noted that incentivizing health professionals to work in rural areas or primary care centers is challenging due to low salaries. Suggestions included incorporating placements in training, offering higher city salaries, continuous education, and encouraging physicians to provide free or discounted services in their hometowns. Some informants noted that many countries face a similar challenge regarding incentivizing rural/primary care work. Addressing the lower social development in such areas may be the solution.

If there is a school in Hermel and you ask the teacher you want to live in Beirut or Hermel, he will say Beirut. Why? Because I will have better life, and a better salary, and I can send my kids to a better school and there is entertainment. So, to go out to rural areas outside the city you have to develop these rural areas […] Some of the activities that were done with the previous ministries had to do with decentralization and giving a stronger role to municipalities. This could be a way to incentivize people to move outside and really enjoy working outside. (P3)

Informants noted that the business model of rural primary care centers is insufficient to incentivize health professionals. Suggestions included involving physicians and nurses in decision-making, improving center structure and governance, and acknowledging that most physicians cannot be adequately compensated in primary care centers. There were also concerns about whether Lebanon has enough doctors for these centers.

### Health information systems

#### Electronic health/medical records

Most informants supported a national electronic health record (EHR) system but were less in favor of electronic medical records (EMR; which are limited to one facility). Cost was seen as a major barrier, with some suggesting delays and others advocating for its efficiency benefits. An alternative suggestion was adopting a medication card, currently being developed by the Order of Pharmacists. Distinctions were also made regarding EHR features.

You cannot access the EMR of each hospital, but you can access EHR that will give you the essential information needed, especially the emergency rooms to minimize duplication. (P9)

Regulated yes, but standardized is not necessary. Standardized implies that they’re going to be looking the same. They don’t have to be the same, they have to be regulated, they have to have certain criteria […]and you can use which ever you want, you will end up with a MAC, or a windows, or an IOS, do whatever you want make it look the way you want, providing it meets certain criteria, communication, interoperability, transmit information from one unit to the other, ease of use, etc. (P3)

#### Information infrastructure and development opportunities

Connecting stakeholders across public and private sectors to improve care coordination and management was highlighted by informants, particularly doctors, pharmacists, hospitals and payers. Informants suggested applications like referrals and electronic prescriptions, which could help regulate medications, such as third-party payers paying directly to pharmacies. A medical identifier for everyone could also aid in strategic purchasing of medications, and prevent over-purchasing.

It has to be done at the lowest level for a start, it has to start from pharmacies and practitioners and upload it to a national data center that is performant. And with practice, people will have the habit, and people will also understand that it is helpful for them. It’s better to go to see a doctor or hospital when your file is complete, when people have access to your history, and so on. And this of course can be used as a management tool that would reduce significantly, abuse and financial mismanagement. (P16)

Some informants expressed frustration over the untapped potential of available information, highlighting the need for better internet infrastructure and security. They also suggested that sharing financial information publicly could improve transparency and accountability. Informants noted that private insurance companies have systems linking physicians and pharmacies for electronic prescriptions, but this is lacking nationally and requires standardized coding across third-party payers.

It has been now 22 years since I came to Lebanon and I still hear about e-government, and since I became a general manager, I attended nearly twenty workshops about that subject. Now, frankly, I no longer go because it’s all just talking. (P27)

You have to do standard registration of the medical coding. We have been teaching that for 20 years […]. You need to impose medical coding. It can be done, but there should be a vision […]. (P9)

### People-centered health system

#### Robust gatekeeping

Informants supported gatekeeping through primary care centers or family physicians for improved cost-effectiveness, but noted that national implementation requires considerable resources, strong quality and more family physicians (or general practitioners), which is challenging in the current context. Informants noted that many people in Lebanon prefer specific physicians, making them resistant to gatekeeping. They emphasized the need for public engagement and preparation. Most informants suggested a formal mechanism to bypass gatekeeping, allowing patients to access services directly by paying, or bypass it when dissatisfied with their gatekeeper or when a condition requires direct access.

[We] have to make sure that the gatekeepers or the family physicians or the centers or others receiving the patients are competent […]. Otherwise, such a system cannot be sustained. (P20)

#### People-centered care

Informants emphasized that the individual should be the focus of information, supported by automated services across primary care, hospitals, medications, and third-party payers. This approach would link various facilities, including municipalities and schools. They also highlighted the need to train health students in people-centered care and involve patient voices at all levels, including institutions, committees, and a national patient association.

There should be a patient-representative body […]. Even when we meet there isn’t a patient representative at all healthcare institutions, and we cannot be in the shoes of the patient. (P8)

Informants stressed the need for improved communication and collaboration among health professionals and with patients. They suggested creating accessible information for all backgrounds, linking financial incentives to patient satisfaction, and enforcing Law 174 on smoking. Some proposed focusing on people-centered care at the local level first, with eventual nationwide expansion, while acknowledging challenges to its implementation.

The obstacle to this is that many stakeholders have an interest in not being people-centered or community-oriented; they have interest in it being fragmented. It gives them more money and power. (P3)

## Discussion

**Fig. 1:**
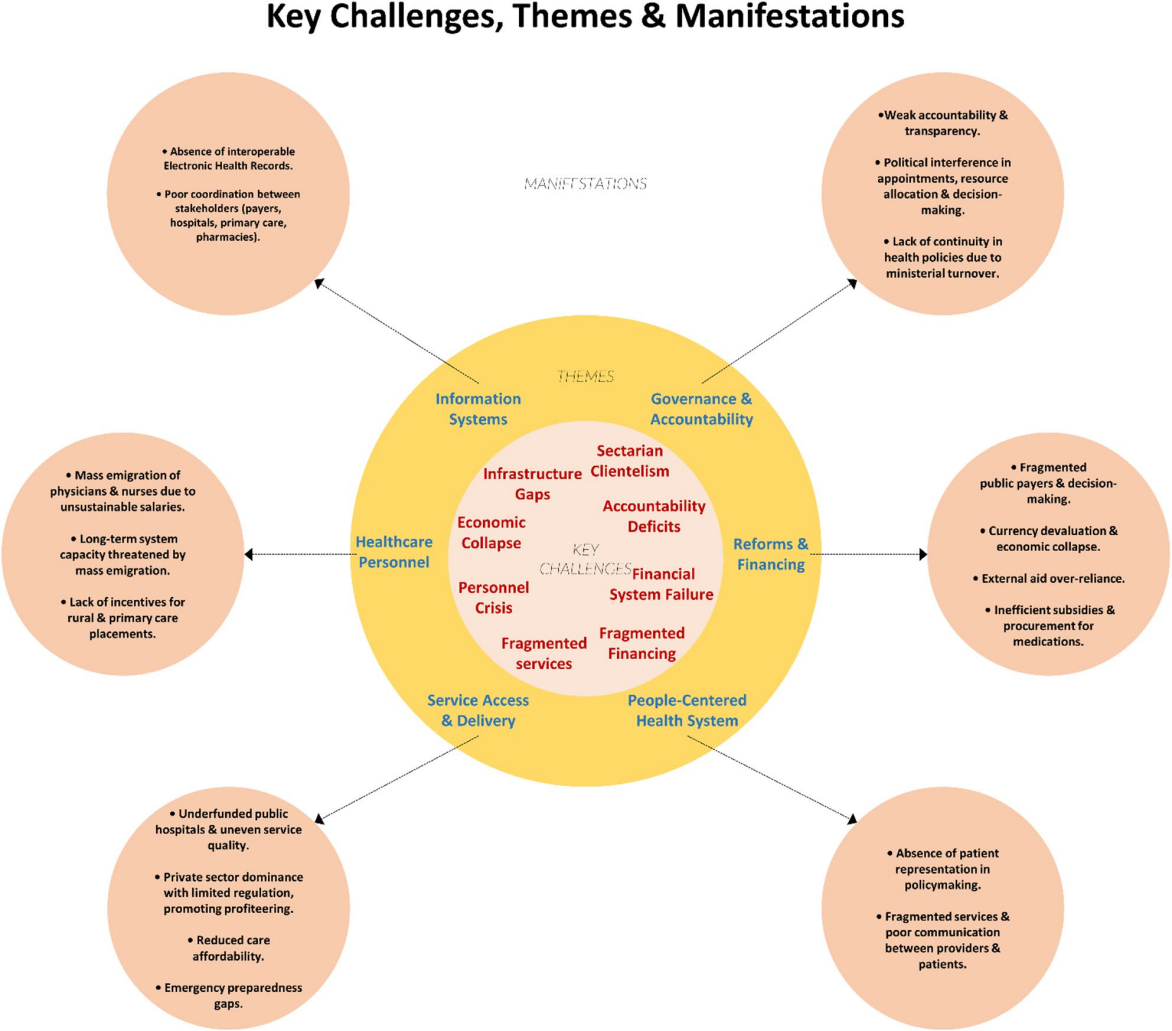
The key challenges of the Lebanese health system, the themes and manifestations they relate to

**Fig. 2:**
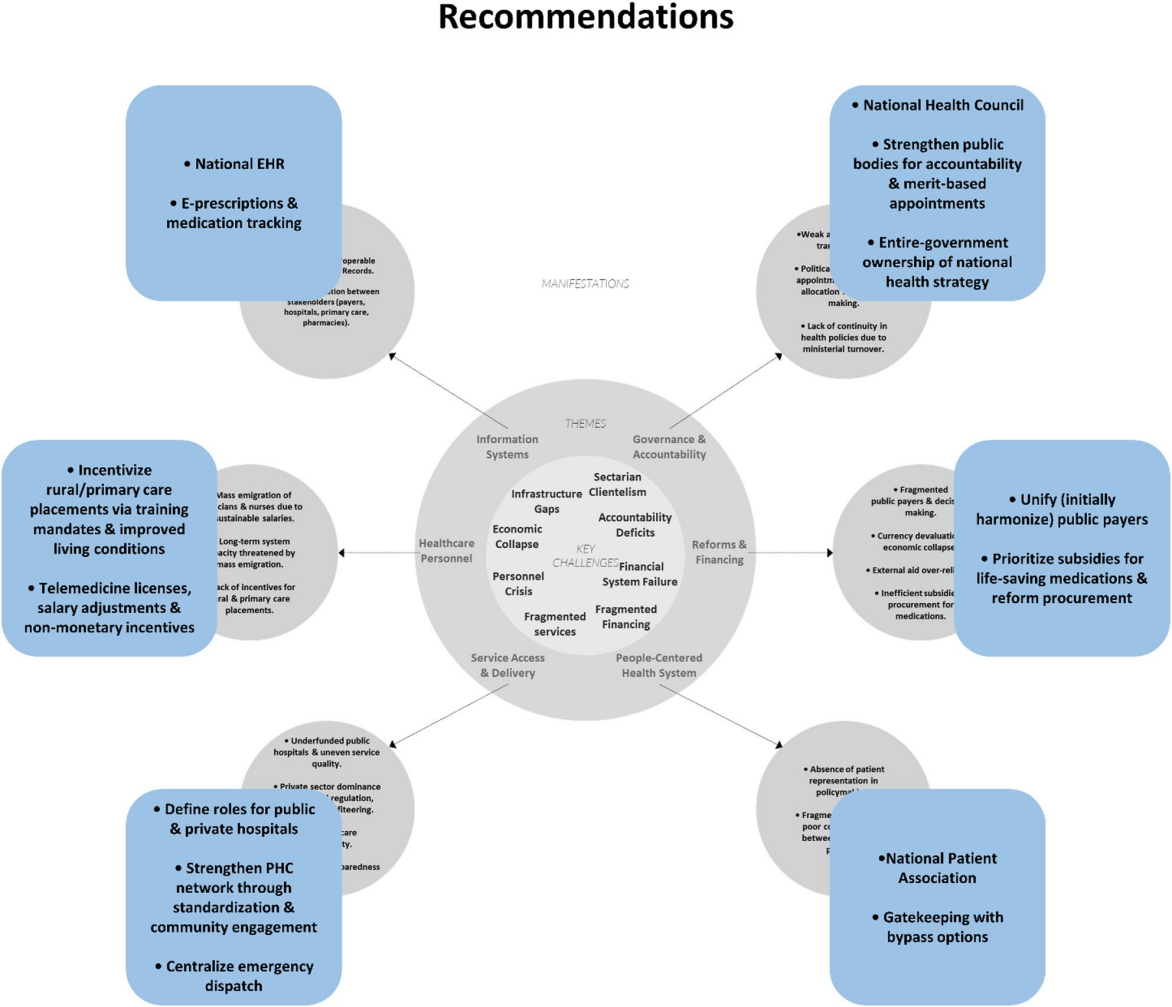
The recommendations for reform and development of the Lebanese health system

The results of this study highlight several interrelated challenges facing Lebanon’s health system, which contribute to its fragility and compromise equitable, efficient care for its population. These challenges are attributable to key challenges plaguing the Lebanese context, including sectarian clientelism, accountability deficits, financial system failure, economic collapse, fragmented financing and services, personnel crisis and infrastructure gaps (see Fig. 1). While the challenges are considerable, the study also identifies past and ongoing successes, as well as actionable recommendations for reform and development (see Fig. 2).

Six key themes emerged from the interviews, encompassing 26 subthemes and addressing fundamental challenges in health system governance, accountability, financing, access and delivery, healthcare personnel, information systems and technology, and people-centered health system. These themes are deeply interconnected, with governance failures emerging as foundational drivers that exacerbate challenges across all other domains. For instance, fragmented financing (theme 2) is not merely a technical issue but a direct consequence of sectarian clientelism, where multiple public payers are maintained to serve political constituencies. This fragmentation, in turn, undermines equitable service delivery (theme 3) by creating unequal coverage and contributing to the financial crisis facing providers. Similarly, the healthcare personnel crisis (theme 4) is primarily driven by the broader economic collapse originating from the banking sector’s Ponzi-like structure and unsustainable fiscal policies, which precipitated the currency devaluation and hyperinflation that have eroded salaries to unsustainable levels and driven mass emigration.

Conversely, strengthening health information systems (theme 5) was identified by informants as a potential mechanism to increase transparency and reduce political interference by enabling data-driven oversight. These interconnections (partially visualized in Fig. 1) suggest that isolated interventions are unlikely to succeed; rather, reforms must simultaneously address governance as the root cause while implementing targeted improvements in financing, workforce and service delivery that can reinforce each other. For system-level understanding, this implies prioritizing governance reforms that enable technical solutions to take root, while recognizing that improvements in information systems and people-centered care can, over time, create accountability pressures that further constrain political interference.

These six themes and 26 subthemes, detailed in the Results section and summarized in Table 2, form the empirical foundation for the recommendations presented in Tables 3 and 4. Table 3 was constructed by systematically mapping each theme and subtheme to corresponding actionable recommendations, drawing on both informant suggestions and evidence from comparable health system reforms in other countries. The most impactful recommendations (italics in Table 3) were selected based on their potential to address root causes and their recurrence across multiple informant accounts. Table 4 further prioritizes these into scalable short-, medium-, and long-term actions based on feasibility, potential impact, and time horizon for implementation in the current Lebanese context.

The following sections discuss these findings in the context of Lebanon’s unique socio-political context, with explicit linkages to global evidence and policy lessons. We also elaborate on specialized topics with current policy implications in Lebanon, including public-private partnerships, emergency preparedness and health security, gatekeeping and people-centered care.

### Governance and accountability

Governance issues, especially those tied to political interference, are at the heart of Lebanon’s health system challenges. The study’s findings reveal that political affiliation with health institutions weakens transparency, disrupts continuity, and undermines the integrity of health reforms. Sectarian clientelism is a major driver of political interference, which in turn enables corruption. Informants pointed out that political actors often influence key decisions, ranging from leadership appointments to resource allocation. These findings are consistent with previous research, which has emphasized that political patronage and weak accountability in healthcare governance erodes the effectiveness of health systems in low- and middle-income countries [17, 18]. In the Lebanese context, patients perceive the negative role of political interference in the healthcare system, including through personal connections and favoritism [19]. These governance failures have tangible health consequences. As informants noted, political interference in medication supply chains and delayed reimbursement decisions have contributed directly to treatment interruptions for cancer patients and increased mortality from delayed hospital presentation - outcomes that reflect systemic accountability deficits rather than resource constraints alone. Recommendations to create independent governance mechanisms and oversight institutions aim to establish accountability as a central pillar of health reform.

Suggestions by informants for a National Health Council or a similar independent regulatory body, and independent audit bodies underscore the importance of separating health sector governance from political interests. This aligns with the work of Savedoff (2011), who argues that health systems in fragile states require autonomous public institutions with strong accountability, to prevent political capture and ensure continuity in health policies [20]. Such a body may also play a major role in the inclusion of the population in a participatory governance approach, and in ensuring the continuity of health strategy across successive governments.

In Thailand, the National Health Commission was developed in 2007 with a mandate to discuss critical policies and implement reform [21]. The NHC had a triangular composition with equal representation of three groups: government technocrats, policy-makers and politicians; civil society, communities and the population; and academia and think tanks. Notably, over half of NHC members did not come from the health sector, thus contributing to a holistic approach to health, and its office is chaired by the Prime Minister rather than Minister of Health “the ideation of health comes from every sector” [21]. In China, a national commission and the Ministry of Health developed a master policy document, and the Health System Reform Office was created to coordinate reform implementation across ministries [22]. 

### Public-private partnerships

Informants were divided regarding whether or not PPPs present a potential avenue for health system strengthening in Lebanon. While some see PPPs as a means to channel private sector expertise and resources, others cautioned against the risk of profiteering and reduced accountability. Informants proposed that effective PPPs should be linked to clear governance structures and transparent agreements that prevent political interference.

There is mixed evidence regarding the effectiveness of PPP for hospital or primary care, perhaps largely due to the varied contexts and complexity of such initiatives. In some instances, PPP may result in improved accessibility, however, the impact on equity, quality and efficiency is uncertain (Liu et al. 2008). Evidence from better regulated contexts such as Australia, Spain and the United Kingdom suggests that PPP may further complicate the already complex task of developing and operating hospitals [23]. Despite the widespread interest in PPP over the past three decades, the limited evidence suggests that more thorough evaluations are required [24, 25].

Given the mixed evidence on PPP effectiveness in healthcare, such initiatives require careful design and a context-specific approach. While PPPs may offer partial solutions in some instances, they should not be relied upon as a major solution to address Lebanon’s health system challenges, particularly given the risks of inequity, cost escalation and inadequate accountability. Beyond PPPs, which entail long-term risk-sharing, more immediate regulatory tools include mandating price transparency for common procedures, standardizing quality reporting across private facilities, and linking licensure renewal to participation in public health reporting. Such measures would balance private sector autonomy with public accountability.

It is relevant to make a distinction between PPP and contracting. Contracting out involves the government paying private entities to deliver specific services, while PPP involve broader, long-term collaborations where the private sector shares in financing, developing and managing infrastructure or services. Contracting for health service delivery has generally been beneficial in developing countries, although experiences are also subject to differences in design and context [26]. In Lebanon, there is a longstanding history of such contracting between public payers with providers of hospital and primary care [27, 28].

### Emergency preparedness and health security

The COVID-19 pandemic exposed significant gaps in Lebanon’s health system preparedness capacity. This specifically applied for the period beyond the initial response, as Lebanon’s strategy was successful in preventing COVID-19 spread throughout the first several months of the pandemic. When containment of the virus eventually failed, informants noted that public hospitals bore the brunt of the response, with private hospitals playing a limited role.

This is consistent with findings from other countries where public health systems were heavily pressured or overwhelmed [29–32]. In some countries, this has led to governments to mandate or incentivize greater private sector involvement. For example, in Malaysia private hospitals were obliged to receive certain categories of COVID-19 patients or otherwise risk fines or jail time, in accordance with its pre-existing Prevention and Control of Infectious Disease Act of 1988 [31]. In Ireland, the Government negotiated an agreement to access the capacity of private hospitals for a period of three months [30]. More broadly, the pandemic has been suggested to have exposed the long-going market and governance failures regarding the private health sector across LMICs [33]. This has led to three specific crises in financial and liquidity crisis among private providers, in service provision and pricing, and in state-provider relations [34]. 

In Lebanon, our findings highlight the need for better coordination of emergency preparedness efforts and a more integrated approach to crisis management. This may also be in the form of a central coordinating body for emergency health services. It is notable that Lebanon’s economic crisis that began in October 2019 would have considerably exacerbated the financial and liquidity crisis for private (and public) hospitals. Establishing legislation outlining public-private collaboration in emergency response such as in some countries would also be beneficial, particularly in the crisis-prone Lebanese context.

Primary health care (PHC) also has a considerable role to play in health emergencies. However, informants noted that primary care centers were largely excluded from the pandemic response. PHC response varied widely across countries, despite early guidance from WHO emphasizing the role of PHC [35]. This was often related to the health system integration of primary care - or the lack thereof. For example, countries that did involve PHC in testing for COVID-19 included Australia, Canada, China, Germany, New Zealand and Singapore; those that did not included Brazil, France, Italy, Iran, Portugal, Spain, the United Kingdom and the United States [36]. Generally, East Asian countries were better prepared, having learned the lessons of the 2003 SARS epidemic [36]. Lessons learned emphasize the need not only to strengthen PHC, but also protect primary care personnel and clearly define the role of PHC in pandemic response [37]. There is also a need to more clearly develop the role of PHC in addressing epidemics [38]. 

### Reform and financing

Financial constraints are one of the most pressing challenges for Lebanon’s health sector. The fragmentation of public payers and inadequate financing structures have limited service provision efficiency and equity. This finding aligns with the broader literature on healthcare financing, which suggests that fragmented funding models tend to perform poorer in terms of health outcomes and cost [39, 40]. Social Health Insurance (SHI) schemes whereby people contribute to a health fund (often mandatory, e.g. Lebanese NSSF) have generally been successful in high-income countries with a large formal sector. However, in low and middle income countries the use of SHI may contribute to further fragmentation and increased inequity, particularly when not all citizens are included. SHI experiences in Ghana, Ethiopia, Kenya, Rwanda and Tanzania suggest such schemes do not allow equity goals to be reached, and may not be effective for reducing out-of-pocket payment (OOP) [41, 42]. To achieve universal coverage, health systems should expand risk pooling, and maximize the integration of financing mechanisms while relying on prepaid plans [39, 40].

Globally, there is a growing trend among countries to reduce the number of public payers as an approach to improve health system efficiency and equity. Notable successful examples include Thailand, Turkey, China, Brazil and Mexico [43–48]. Thailand consolidated its three major public payers into one in 2001, under its Universal Coverage Scheme [47]. In 2006, Turkey merged its three major public payers—one for private sector workers, another for civil servants and retirees, and a third for the self-employed—into a single entity, the Social Security Institution, reaching near-universal health insurance for its population [43]. In 2016, China successfully consolidated its health insurance system for its 1.4 billion population, from three payers to two by merging the urban and rural resident schemes, to improve administrative efficiency [44, 48].

In our study, some informants support the unification of public payers, while others view harmonization as a more feasible short-term goal. However, such reforms would require significant political will, which is currently in short supply in Lebanon. Nevertheless, the unification of payers could streamline administrative processes, improve cost control and enhance purchasing power in negotiations with providers. Informants emphasized that health financing reform must address the broader macroeconomic crisis, financial accountability and explore alternative revenue generation mechanisms. The latter may include increased taxes on harmful products (e.g. tobacco and alcohol), but is not expected to have a large-scale impact.

This reflects a broader tension in the findings: while unification would yield long-term efficiency gains, informants emphasized that in the immediate term, harmonization (aligning reimbursement rates, procurement, and information systems across payers) offers a more politically and logistically feasible pathway that can begin to reduce fragmentation without requiring full structural consolidation during an acute crisis.

Over-reliance on external aid was another key issue raised by the informants, who also questioned the long-term sustainability and over-reliance on external aid for primary care centers in particular. While international funding has been crucial in sustaining Lebanon’s healthcare system during times of crisis, this model is not sustainable in the long term. External aid is often unpredictable, and it can create dependencies that weaken the system’s ability to develop its own priorities and long-term strategy [49, 50]. Path dependence may also result in structures that hinder reform, as institutions persist for extended periods not because they are the most effective, but because altering established norms and processes is costly [51]. This may be particularly detrimental when the system targets select groups rather than the broader population, possibly resulting in the displacement of local health needs.

Multiple donor funded projects also necessitate greater coordination. The absence of such coordination results in overlapping initiatives, inefficient resource use and service gaps, especially in low-resource settings with limited local capacity [49]. The eventual withdrawal of aid-funded programs may leave communities without the essential services those programs once provided [52]. 

Ammar et al. (2016) noted that Lebanon received inconsistent and inadequate external aid during the Syrian refugee crisis, failing to meet refugees’ health needs [7]. Despite this, healthcare services for both citizens and refugees were sustained, largely through collaboration between the Ministry of Public Health, UN agencies, and NGOs. However, the system experienced significant strain, and citizens perceived that refugees were prioritized over them, resulting in fragmented coverage and potential resource displacement [19]. This fragmentation likely weakened the healthcare response and exacerbated poor health outcomes during the economic crisis that emerged in 2019.

Prioritizing a sustainable, locally financed healthcare system is essential, especially as international aid may decline due to geopolitical changes or donor fatigue. A glaring example is given by precipitous decrease of international aid from the US in early 2025, impacting numerous countries. In Lebanon, resolving the Syrian refugee crisis is equally critical, not only for the well-being of refugees but also for the long-term sustainability of the health system.

Aside from the over-reliance on external aid, informants widely recognized the success of the collaborative approach to the national primary care network, and in particular the role of community-minded leaders. Despite the additional burdens due to the refugee influx and subsequent economic crisis, developing the network should remain a priority for the health system, and resources should be dedicated from the State towards this. Variation in service quality and the lack of financial transparency were noted as limitations by informants.

### Healthcare access and delivery

Informants considered public hospitals to be crucial, and that their role should be defined, also in relation to private hospitals. However, they considered that public hospitals were underfunded and overburdened, limiting their capacity to deliver high-quality care. While they were central to crisis response, they were often under-resourced during COVID-19 and absent in the Beirut port explosion response. Better collaboration is needed with private hospitals, although these had a very limited role during COVID-19. Most informants disagreed with limiting public hospitals to low-income populations, and emphasized that private hospitals also share responsibility for caring for vulnerable groups. The timely reimbursement of private hospitals by public payers and in particular by the Central Bank is a major factor for ensuring sustainability and trust among healthcare stakeholders.

In the short term, strengthening public hospitals requires not only capital investment but also operational measures: expediting overdue reimbursements to ensure liquidity, establishing formal referral pathways between public hospitals to distribute patient loads, and deploying mobile health units from better-resourced facilities to underperforming ones during acute surges.

Informants viewed the service package as generous but suggested improving efficiency through better fund allocation, rather than reducing it. They agreed on retaining primary, hospital, and chronic care services, including medication, to support vulnerable populations and equity. Recommendations included monitoring outcomes, excluding some costly medications, empowering the MoPH medications committee and training physicians to safeguard the system. They also proposed condition-based eligibility, identifying all covered individuals and clarifying expatriate coverage.

The 2019 economic crisis had a major impact in decreasing healthcare accessibility, as most people did not have access neither to their bank savings nor sufficient new hard currency. This was further compounded by public payers using a lower currency exchange rate to pay a fraction of actual costs to hospitals, with hospitals in turn requesting higher out of pocket payment from patients. The impact of the crisis was reflected in decreased utilization of healthcare, and eventual worsening of health outcomes. The 2023 multi-sector needs assessment showed that 92% of interviewed Lebanese households reported being unable to meet al.l their essential needs, with the top reported need being health care as reported by 64% of the interviewees [53]. 

Informants highly valued the roles of stakeholders in emergencies, particular the Lebanese Red Cross, civil service and select hospitals. However, they noted that the current capacity is insufficient. Integrated emergency services were suggested as a solution by some informants, similar to the SAMU in France. However, given the Lebanese context and high resources required for a SAMU-like solution, Lebanon may benefit most from a centralized emergency management system that integrates public and private healthcare resources, with paramedic-led pre-hospital care (rather than physician-led). It would also rely on community volunteers for rural coverage, and use cost-effective technology for coordination. Such a system would be similar to those in place in countries such as Brazil, Ghana, Malaysia, Mexico and Thailand.

Another major issue raised was access to medications, which remains a persistent problem, particularly for lower-income populations. Lebanon’s economic crisis has had a devastating impact resulting in medication shortages and making it increasingly difficult for patients to access life-saving drugs. This is consistent with findings from other countries that had experienced such crises, where medication shortages have led to increased morbidity and mortality [54]. In Venezuela, the economic crisis has similarly disrupted medication supply chains, leading to widespread shortages and increased mortality rates [55]. 

In Lebanon, the crisis exposed the fragility of the health system, particularly the lack of a robust supply chain and the over-reliance on imports whose volume exceeds local medications more than ten-fold. Supply chain improvements and reforms in regulation and pricing would be beneficial, but perhaps even greater benefits may be realized with the adoption of electronic prescription and subsidization on the basis of the individual rather than the medication.

### Healthcare personnel

Healthcare personnel challenges, especially the retention of physicians and nurses, are among the most critical issues raised by informants. The economic crisis has reduced salaries to unsustainable levels, driving health professionals to emigrate. Such ‘brain drain’ is not a new phenomenon, and is also fueled by high-income countries’ recruitment policies that benefit these countries at the expense of LMICs that have invested scare resources to train health professionals [56]. However, an economic crisis increases brain drain to an unprecedented scale.

It is unsurprising that financial compensation is the major factor, similar to that seen in other countries that have faced such crises, such as nearby Greece [57]. Improving compensation is a necessary albeit challenging intervention, but other measures should also be explored, such as promoting job satisfaction, professional development, and non-monetary incentives [58]. Short-term consultations within or outside Lebanon may be an alleviating measure particularly for physicians, and is currently being practiced by Lebanese with neighboring countries [56]. It may also be advisable for the Lebanese State to develop a formal response, similar to Thailand’s use of supply and demand-side interventions following its economic crisis two decades ago [59]. 

In addition, rural places typically have greater health disparities, and efforts to strengthen rural placements and primary care roles require greater socioeconomic development in rural areas, as well as incentives for personnel. While there are no clearly set solutions to such a challenge, in LMICs this may be best addressed with intervention bundles including attention to living environments, working conditions and development opportunities [60]. 

### Health information systems

The development of health information systems and the use of electronic health records (EHRs) are seen by informants as essential for health system reform, which aligns with global trends. Informants emphasized the need for interoperability and data flow between different health sector actors, including public and private payers, hospitals, pharmacies, and providers. Some informants emphasized the distinction between EHRs and electronic medical records (EMRs). EMR is a digital record of a patient’s medical history within one organization, while EHR is a broader system that shares patient information across providers, ensuring accessibility and interoperability without requiring standardization.

While there is a potential for EHRs to improve care quality, patient safety and cost, studies have had mixed findings due to the heterogeneity of systems and study designs, and any initiatives should consider both positive and negative effects [61, 62]. Poorly designed EHR systems may worsen care quality and jeopardize patient safety, and may result in physician attention being shifted towards data entry and away from patients [63, 64]. In limited resource settings such systems may not be scalable due to constraints in human resources and cost [65]. It is necessary to emphasize that EHR systems be well-designed and properly implemented [66]. In Lebanon, the Ministry of Public Health has made use of its limited EHR for designing, implementing and evaluating policy interventions, although other public payers lack such a system [67, 68]. Nevertheless, developing nationwide EHR covering all healthcare interactions (including emergency visits) is necessary for health system regulation and development.

Informants also highlighted the importance of having a unique medical identifier to better regulate medication purchasing and allow strategic purchasing. However, they also noted the need to ensure better internet infrastructure and security.

### Gatekeeping and people-centered care

Informants considered that gate-keeping through primary care centers or family physicians was an important measure to improve system cost-effectiveness. However, this should be accompanied with a bypass mechanism for use in specific circumstances, as well as ensuring high-quality gatekeepers and public engagement. Gatekeeping is a commonly a topic for health system reforms. However, the evidence on the impact of gatekeeping is of limited quality [69]. Gatekeeping is associated with lower healthcare utilization and expenditure and improved care quality, but is also associated with lower cancer survival and lower patient satisfaction [70]. Therefore, system design and context should be carefully considered.

Gatekeeping in healthcare varies globally: some countries such as the UK and Thailand enforce strict systems requiring primary care contact before specialist access, while others such as Singapore and Brazil use financial incentives and public education to encourage primary care use without strict mandates. In the Lebanese context, many patients ‘follow their doctor’ [19]. Considering this and other contextual factors, a non-strict gatekeeping approach would be more appropriate in Lebanon.

People-centered care emerged as a cross-cutting theme, with informants calling for patient representation in decision-making, as well as the creation of a national patient association. Health systems can benefit in various ways from a wider engagement of patients [19]. Informants also emphasized the need to improve information sharing with patients. Patients in Lebanon place high importance on the need for clear information, and more broadly they desire to be treated with dignity and respect [19]. Ensuring communication and engagement is essential for improving health equity and ensuring that reforms reflect public needs.

In recent decades, many countries have become increasingly aware of the need to focus on the patient’s role, and at the 69th World Health Assembly (2016) the Framework on Integrated People-centered Health Services was adopted by member states, highlighting the patient role in defining their needs and in co-producing health services [71]. A people-centered health system should ensure that health professionals are humane, informative and not money-driven [19]. 

Synthesizing the global lessons across themes, several patterns emerge. On governance, the experiences of Thailand and China demonstrate that independent oversight bodies and cross-ministerial coordination can sustain reform momentum beyond political cycles. On financing, Turkey and China illustrate the feasibility of payer consolidation for administrative efficiency, while Ghana and Tanzania caution that social health insurance alone may not achieve equity without broader risk pooling. On emergency preparedness, Malaysia and Ireland show that legislative frameworks for public-private collaboration are critical for crisis response, while East Asian countries’ integration of primary care into pandemic response underscores the importance of pre-established protocols. On workforce, Thailand’s post-crisis interventions combining supply and demand-side measures offer a model for mitigating brain drain. These lessons reinforce that context-specific adaptation, rather than wholesale adoption, is essential, and that sequencing (e.g. harmonization before unification) is as important as the reform itself.

**Fig. 3:**
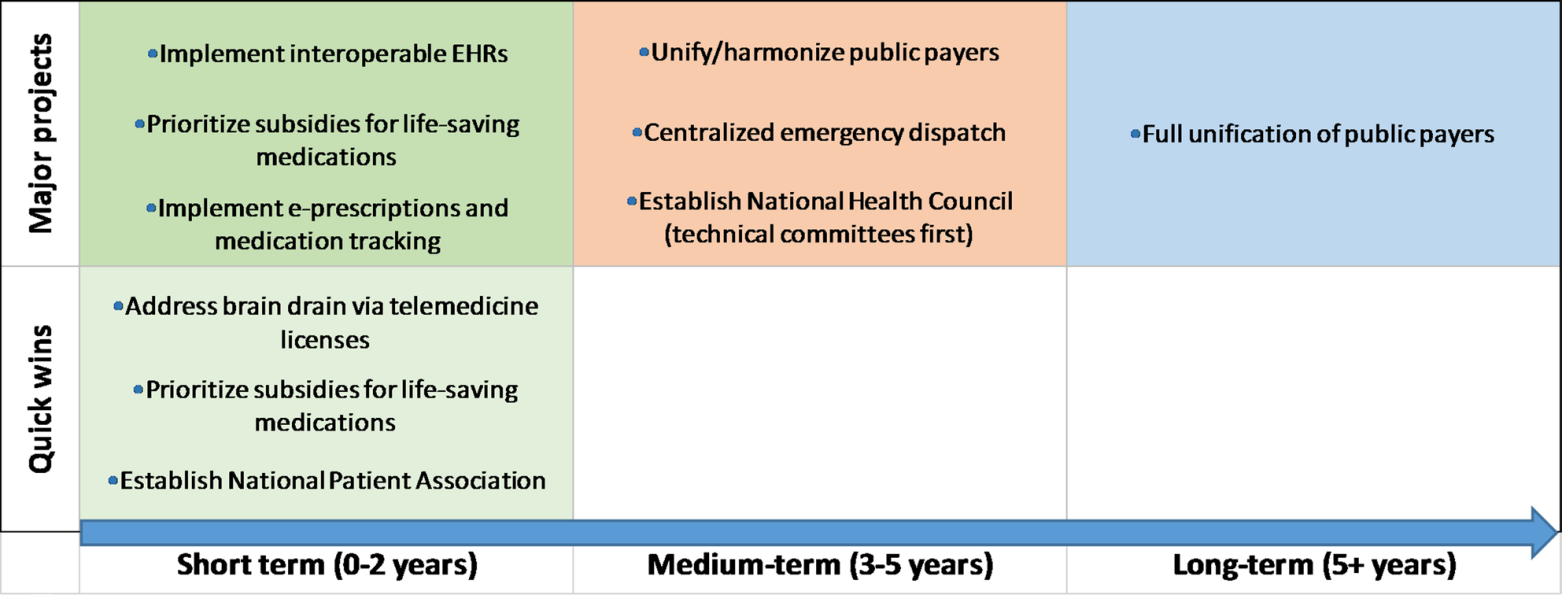
Most scalable recommendations across timeline

Based on the study’s findings and the review of the literature, we have developed a list of recommendations for the Lebanese health system. Table 3 was constructed by systematically mapping each theme and subtheme identified in the analysis to corresponding actionable recommendations, drawing on both informant suggestions and evidence from comparable health system reforms in other countries. Recommendations were iteratively reviewed by the research team to ensure they addressed the core challenges identified while remaining contextually appropriate for Lebanon. Table 4 was then developed by the research team through consensus-based prioritization, assessing each recommendation against criteria of feasibility, potential impact, and time horizon for implementation in the current Lebanese context. The most impactful recommendations (italics in Table 3) were selected based on their potential to address root causes and their recurrence across multiple informant accounts. These are detailed in Table 3, and illustrate most of these in Fig. 2. We also present the most scalable recommendations by timeline in Table 4, and illustrated in Fig. 3.

**Table 3:** Recommendations based on study findings, with the most impactful being italics

Theme/Subtheme	Recommendations
Governance & Accountability	*1. Establish an independent oversight body (e.g. National Health Council).* *2. Ensure entire-government ownership of national health strategy.* 3. Strengthen public bodies (e.g. Central Inspection, Audit Bureau) to enforce accountability and merit-based appointments.
Health Financing	*4. Unify (initially harmonize) public payers to streamline financing.* *5. Prioritize subsidies for life-saving medications*,* encourage use of generic medications*,* support local production and reform procurement.* 6. Explore sustainable local financing (e.g. taxes on tobacco, alcohol).
Service Delivery & Access	*7. Define clear roles for public and private hospitals.* *8. Develop a centralized emergency response system (paramedic-led*,* with public-private coordination).* 9. Strengthen primary healthcare networks through standardization and community engagement.
Healthcare Workforce	*10. Address brain drain via telemedicine licenses*,* salary adjustments*,* and non-monetary incentives.* 11. Incentivize rural and primary care placements via training mandates and improved living conditions in host community.
Health Information Systems	*12. Implement interoperable electronic health records (EHRs).* *13. Implement e-prescriptions and medication tracking.* 14. Invest in digital infrastructure (e.g. internet security, standardized coding).
People-Centered Reforms	15. Adopt a non-strict gatekeeping model and ensure competent gatekeepers. *16. Create a national patient association to amplify patient voices in policymaking.*
Public-Private Collaboration	17. Regulate private sector engagement through clear contracts and performance benchmarks. 18. Use context-specific contracting (not blanket PPPs) for discrete services.
Crisis Preparedness	19. Legislate mandatory private sector involvement in emergencies (e.g. pandemics). 20. Integrate primary care into emergency responses (e.g. testing, vaccination).
Cross-Cutting Priorities	21. Address political interference via legal safeguards and public awareness campaigns. 22. Reduce reliance on external aid, while coordinating ongoing aid to align with national priorities and avoid duplication.

**Table 4:** Select recommendations considered to be the most scalable

Timeline	Recommendation	Rationale for Scalability	Expected Impact
Short-Term (0–2 years)	Address brain drain via telemedicine licenses	Utilizes diaspora doctors without requiring salary hikes; minimal legal/tech barriers.	Retains specialist access while mitigating workforce losses.
	Prioritize subsidies for life-saving medications	Targets limited funds to critical drugs; support local production and e-prescriptions for rapid implementation.	Immediate reduction in mortality from treatable conditions.
	Implement e-prescriptions and medication tracking	Uses existing pharmacy networks; low-cost digital solutions (e.g. mobile apps).	Reduces medication shortages/errors; curbs black-market sales.
	Implement interoperable EHRs	Uses open-source platforms and existing hospital IT systems; phased expansion.	Improves care coordination, reduces duplicate tests/medication errors.
	Establish National Patient Association	Builds on existing advocacy groups; low-cost civic engagement model.	Amplifies patient voices in policymaking and safeguards against political interference
Medium-Term (3–5 years)	Unify/harmonize public payers	Start with reimbursement alignment (e.g. standardized fees), delaying full merger.	Cuts administrative waste, improves equity in service coverage.
	Establish National Health Council (Technical committees first)	Avoids political gridlock by starting with expert-led working groups.	Depoliticizes decision-making, ensures reform continuity.
	Centralized emergency dispatch	Integrates existing Lebanese Red Cross infrastructure; avoids building from scratch.	Saves lives in crises through faster coordination.
Long-Term (5 + years)	Full unification of public payers	Requires prior success in harmonization and political consensus.	Creates sustainable, equitable financing system.

### Limitations

This study has limitations that should be acknowledged. First, while purposive sampling ensured the inclusion of diverse and knowledgeable stakeholders across the Lebanese health system landscape, the selection of key informants may have been influenced by the research team’s professional networks and longstanding engagement within the sector. Although we explicitly sought to represent institutional hierarchies and health system functions, some perspectives, particularly from frontline clinical staff without formal leadership roles or from patients themselves, may be underrepresented. To mitigate this, we ensured that the sample included voices from primary care centers, nursing and patient association leadership, but we acknowledge that a broader inclusion of non-leadership staff and direct patient experiences could have enriched the findings further.

Second, the specificity of this study to the Lebanese context strengthens the study’s contribution to understanding health systems in fragile settings, it also limits the direct generalizability of findings to more stable health systems. Nevertheless, as noted in the Introduction, Lebanon represents an extreme test case, and the lessons regarding reform sequencing, governance insulation, and workforce retention strategies may offer valuable insights for other resource-constrained settings facing compound shocks.

Third, the interviews were conducted in 2022, prior to the escalation of conflict in October 2023 and again in March 2026. While this timing captures the health system at the peak of the economic and institutional collapse, the subsequent conflict has inflicted additional damage on health infrastructure and caused further displacement. However, as noted in the Introduction, the preliminary findings of this study informed the National Health Strategy: Vision 2030, and the core systemic vulnerabilities identified remain highly relevant. The conflict has only intensified the urgency of the reforms proposed herein.

## Conclusion

The findings of this study underscore the urgent need for transformative reforms for Lebanon’s health system. Given its multiple ongoing crises, ensuring essential services is critical, but more fundamental reforms are necessary to address the key challenges and their manifestations. Delay in taking critical choices for health system recovery would exacerbate population harm.

We distinguish two interdependent reform horizons. Immediate stabilization measures (0–2 years) can be implemented with existing resources and political will, including e-prescriptions, telemedicine licenses, centralized emergency dispatch, interoperable electronic health records, and a National Patient Association. These measures address the most pressing access and workforce challenges while building momentum for deeper reform. Foundational structural reforms (3–10 years) require sustained political consensus but are essential for long-term sustainability, and include harmonization then unification of public payers, a National Health Council with technical leadership, defined roles for public and private hospitals, and improved working conditions for health personnel.

Achieving these reforms hinges on separating health governance from political interference and empowering technical leadership. To this end, stakeholder dialogue should move beyond ministerial-level negotiations to structured platforms that include civil society, professional syndicates, and patient representatives, such as the proposed National Health Council, to build accountability and ensure continuity across governments.

As Lebanon’s crisis represents an extreme test case for health system sustainability, the solutions explored here, particularly the sequencing of harmonization before unification, the use of telemedicine to mitigate workforce losses, and the role of independent oversight bodies in fragile contexts, may inform recovery efforts in other resource-constrained settings facing compound shocks.

## Electronic Supplementary Material

Below is the link to the electronic supplementary material.

Supplementary Material 1

## Data Availability

The written transcripts of key informant interviews are available in anonymized format upon reasonable request from the corresponding author.
