# Supplementary material for: Bridging crisis recovery and long-term transformation of the health system in Lebanon: evidence from key informant interviews and global lessons
Source: BMC Health Serv Res. 2026 Apr 27;26:828. doi: 10.1186/s12913-026-14630-y (PMC13270591; doi:10.1186/s12913-026-14630-y)
Supplement: Supplementary file 1 — Supplementary Material 1 [file 12913_2026_14630_MOESM1_ESM.docx]

**­­Lebanon Health Sector Strategy Development**

**Interview guide for key informant interviews**

# **Provision**

1. Do you agree or disagree with the following statement:

“Except in emergencies, it is still rational and affordable (for both public payers and patients) to have immediate access to tertiary healthcare as before (i.e., no waiting lists, and without serious restrictions), including sophisticated hospital care (e.g., orthopedic prosthesis, open heart surgery)”.

Yes No

1.2 Do you have other comments on this issue? Please elaborate [unstructured]

1. Do you agree or disagree with the following statement:

“Except in emergencies, people covered by public payers should have access to the health system only through one gatekeeper of their choice (i.e. family physician/primary care center/public hospital OPD), to receive proper care and then be referred if needed to specialized doctors or hospitals. Those who wish to bypass the gatekeeping and referral system, have to pay for the service.”

Yes No

2.2 Do you have other comments on this issue? Please elaborate [unstructured]

1. Do you agree or disagree with the following statement:

“All public and private institutions should be required to use a standardized and regulated Electronic Medical Record as a tool to rationalize, improve quality and ensure continuity of health care.”

Yes No

3.2 Do you have other comments on this issue? Please elaborate [unstructured]

1. How do you think health services could be transformed towards people-centered care (definition)?

[Open-ended]

1. Beneficiaries of public funds and MOPH were eligible to a generous package of services including sophisticated non-cost-effective ones (such as very expensive last generation drugs).
   Given current financial constraints, what do you think a more realistic/affordable benefit package should include, while remaining acceptable to the population?

[Open-ended]

1. Dealing with emergency cases has been one of the major dysfunctions of the health system. How do you think this should be dealt with?

[Open-ended]

1. Are there any other major issues related to provision of and access to healthcare that you would like to highlight and elaborate on?

[Open-ended]

# **Public sector**

1. Do you agree or disagree with the following statement:

“The Ministry of Public Health should be above all other functions; responsible to ensure heath security and other essential public health functions (examples), in collaboration with other ministries, the private sector and municipalities.”

Yes No

8.2 Do you have other comments on this issue? Please elaborate [unstructured]

1. Do you agree or disagree with the following statement:

“Public hospitals have demonstrated they are indispensable in case of outbreaks and emergencies.”

Yes No

1. Do you agree or disagree with the following statement: “Public hospitals are needed to improve access of the poor to hospital care."

Yes No

1. Do you have other thoughts on public hospitals other than those mentioned in the previous two questions? please elaborate

[Open-ended]

1. How do you think the public sector could be strengthened, considering the political, social, and economic context?

[Open-ended]

1. Is there any other major issue regarding the public sector that you would like to highlight and elaborate on?

[Open-ended]

# **Private sector**

1. Do you agree or disagree with the following statement:

“Since private hospitals are facing tremendous challenges, and some of them are threatened with closure due to the prevailing financial crisis, they should get financial support from the government.”

Yes No

14.2 Other comments on this issue? Please elaborate [unstructured]

1. Do you agree or disagree with the following statement:

“Lebanon should aim to regain its role as a hub for the region (‘مستشفى الشرق’) [use as probing information: hospitals’ great need for hard currency, and taking into account their competitive position in the region in terms of cost and quality; is still desirable, realistic, and feasible.]”

Yes No

15.2 Other comments on this issue? Please elaborate [unstructured]

1. The MOPH has been contracting with the private for-profit sector to ensure hospital care and medicines to its eligible population. How do you think the Public-Private Partnership could be done differently, to become more effective and equitable?

[Open-ended]

1. The MOPH has been mostly relying on NGOs to provide PHC through a nation-wide network of PHC centers, in a framework of collaborative governance involving stakeholders, including municipalities. How can this be maintained or changed?

[Open-ended]

1. Is there any other important issue regarding the private sector that you would like to highlight and elaborate on?

[Open-ended]

# **Financing**

1. Do you agree or disagree with the following statement:

“All the public funds (NSSF, CSC, Military schemes) in addition to the MOPH fund (as insurer), should be unified under one autonomous authority.” [probe on feasibility incl. law amendments, and timing]

Yes No

1. Do you agree or disagree with the following statement:

“Public funds could be kept separate entities in the current situation with the Ministry of Public Health as insurer of last resort. However, their work should be harmonized, including having a unified benefit package.” [probe on feasibility incl. law amendments, and timing]

Yes No

1. Do you suggest other alternatives than in Q.19 and 20? please elaborate [unstructured]
2. Do you think it is possible to undertake major financing reform, under the current circumstances, including law amendments? If so, how and in what direction? [probe regarding major reform versus feasible targeted improvements]

[Open-ended]

1. Are there any additional issues regarding financing that you would like to highlight and elaborate on?

[Open-ended]

# **Human resources**

1. How do you think nurses could be motivated to remain in Lebanon? [probe on market need, dual practice, retention strategy, pension, security, career development]

[Open-ended]

1. Do you think it is important to limit physicians’ emigration? If yes, then how?

[Open-ended]

1. How do you think we could incentivize health workers, including doctors and nurses, to work at primary care centers, in public hospitals, and in rural areas?

[Open-ended]

1. Are there any additional issues regarding human resources that you would like to mention and elaborate on?

[Open-ended]

# **Governance**

1. How do you think one could limit or counter political interference in the health sector? [probe on populistic decisions, favoritism and clientelism]

[Open-ended]

1. How do you think accountability of the public sector, private-for-profit institutions and NGOs operating in the health and social sectors could be improved?

[Open-ended]

1. In your opinion; what other governance issues should be dealt with and how? (unstructured)

# **Other**

1. How do you think the accessibility and availability of medicines could be improved? [probe regarding acute, chronic, catastrophic medications within and outside the MOPH list]

[Open-ended]

1. How do you think we could improve information management and data flow related to health?

[Open-ended]

1. How do you think we could improve health security, including COVID-19, in Lebanon?

[Open-ended]

1. What major challenges do you/did you face in your professional role(s) since 2019?

[Open-ended]

1. Before we conclude this interview, are there any further issues that you would like to mention or elaborate on?

[Open-ended]
